# Dazzle Camouflage Affects Speed Perception

**DOI:** 10.1371/journal.pone.0020233

**Published:** 2011-06-01

**Authors:** Nicholas E. Scott-Samuel, Roland Baddeley, Chloe E. Palmer, Innes C. Cuthill

**Affiliations:** 1 Experimental Psychology, University of Bristol, Bristol, United Kingdom; 2 Biological Sciences, University of Bristol, Bristol, United Kingdom; Istituto di Neuroscienze, Italy

## Abstract

Movement is the enemy of camouflage: most attempts at concealment are disrupted by motion of the target. Faced with this problem, navies in both World Wars in the twentieth century painted their warships with high contrast geometric patterns: so-called “dazzle camouflage”. Rather than attempting to hide individual units, it was claimed that this patterning would disrupt the perception of their range, heading, size, shape and speed, and hence reduce losses from, in particular, torpedo attacks by submarines. Similar arguments had been advanced earlier for biological camouflage. Whilst there are good reasons to believe that most of these perceptual distortions may have occurred, there is no evidence for the last claim: changing perceived speed. Here we show that dazzle patterns can distort speed perception, and that this effect is greatest at high speeds. The effect should obtain in predators launching ballistic attacks against rapidly moving prey, or modern, low-tech battlefields where handheld weapons are fired from short ranges against moving vehicles. In the latter case, we demonstrate that in a typical situation involving an RPG7 attack on a Land Rover the reduction in perceived speed is sufficient to make the grenade miss where it was aimed by about a metre, which could be the difference between survival or not for the occupants of the vehicle.

## Introduction

The term camouflage most often conjures up ideas of invisibility: an attempt to prevent detection of a target. This is, indeed, commonly found among both evolved and man-made attempts at concealment, and is generally achieved by so-called background matching: where the colours and patterns of the target sample those in the environment [1]–[3]. But there are two major problems with this strategy. Firstly, camouflage which works well against one background may not be effective against another [4]–[6]; in other words, there will be strong constraints on both an item's location (as well as the viewpoint of any predator) if it is to remain hidden. Secondly, and more importantly, motion will break this sort of camouflage (*e.g.* [7]): an object that is perfectly concealed when static becomes instantly visible once it starts to move.

However, concealment is only one possible approach to camouflage. If an object is undetectable, then clearly it will be safe. But so too will be an object that doesn't look like the intended target, or that is difficult to localise and therefore capture or hit. It is possible to avoid identification either by making an object look like something else (mimicry or masquerade), or distorting the appearance of that object *via* disruptive camouflage [2], [3], [8].

**Figure 1:**
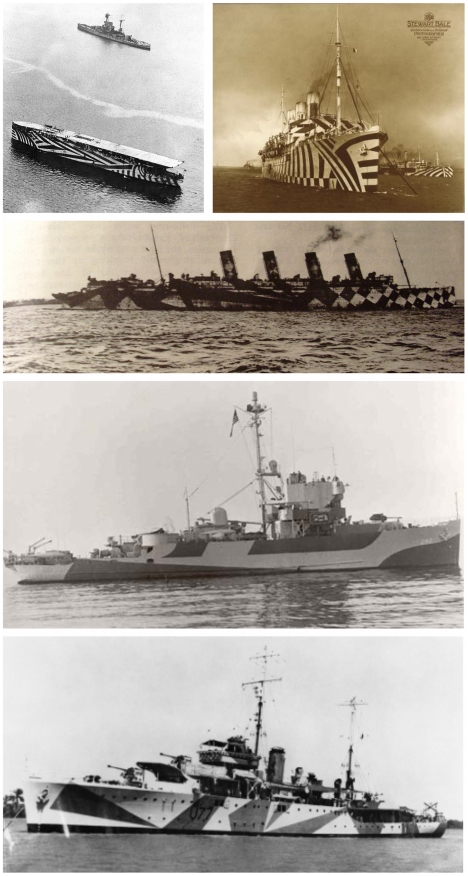
Examples of dazzle camouflage used in the First and Second World Wars. From top left: HMS Argus, SS Empress of Russia, Mauretania (World War One); USS Inaugural, HMAS Yarra (World War Two). Photographs from Wikipedia Commons.

But even if an object has been both detected and identified, it may still be able to avoid capture by distorting its apparent velocity or range, an idea originating with Thayer's [8] theories of animal camouflage but apparently developed independently for military purposes by Wilkinson [9]–[11]. Patterns which aimed to do this were applied to ships during the two World Wars of the twentieth century (see fig.1); the technique became known as “dazzle camouflage” on one side of the Atlantic, and “razzle dazzle” on the other. The claim at the time was that this camouflage would disguise the range, heading, size, shape and speed of individual units, and also make individuating ships difficult if several were sailing together; the primary aim was to reduce losses from torpedo attacks by submarines.

Range finding for targets within visual contact was accomplished using triangulation devices, which relied on combining two images acquired from different horizontal locations (thus allowing the recovery of range using trigonometry). Such combination is harder with any camouflage pattern with repeating elements - it is difficult to work out which parts of each image correspond – and so the calculated distance to the target would be unreliable. Although this method of range finding became obsolete, dazzle markings could still be effective by interfering with perception of trajectory, size and speed. We consider these in turn.

In order to distort perceived heading (direction of travel), camouflage patterns were used which made use of a pictorial depth cue: the texture gradient. This is essentially a richer version of size constancy, the idea that if two objects are known to be the same size, but one looks smaller than the other, then that object is assumed to be further away from the observer. The application of a repetitive pattern which changes its spatial scale along the length of an object gives the impression that the more coarsely patterned end is closer than the more finely patterned one; hence the putative distortion of a camouflaged ship's heading.

Stripes can distort the perception of size, an idea that goes back at least as far as the 19^th^ century (the “Helmholtz square” illusion, in which a horizontally-striped square appears taller and narrower than a vertically-striped one). More recently, different groups have reported variable effects (*e.g.* [12], [13]), with the explicit three-dimensionality of the striped item apparently a critical factor. Regardless of the direction of the effect, all authors claim a size distortion of some sort derived from the application of stripes to an object's surface. Thus it is the case that many dazzle-type patterns might be expected to distort size.

The perception of speed is affected by many disparate factors. For example, larger objects appear to move more slowly than smaller objects [14]; changes in contrast alter perceived speed [15]; differently oriented textures can be seen as moving at different speeds [16]. Any of these effects could be elicited by dazzle patterning.

It is important to note that the large variety of types of dazzle patterns means that the various different distortions outlined above might not all be elicited by a single given pattern; rather, different patterns may be optimal for different types of distortion.

There has been little research into the effects (if any) of dazzle camouflage. To date, evidence of its military usefulness is largely restricted to anecdotal reports (*e.g.* [17]), and the British Admiralty's 1918 analysis of naval shipping losses in WWI was inconclusive, with changes in colour schemes on ships confounded with ship size and other changes in military practice during the war [9], [11]. The only experimental study, motivated not by military camouflage but by Thayer's [8] theories to explain putative dazzle coloration in nature, is [18]: quantifying the capture success of humans searching for variously patterned snake-like targets in a video game, the study did not reveal any systematic advantage of dazzle camouflage over plain coloration. Here, we focus not on capture success, which is the aggregate outcome of different perceptual effects and motor response, but instead test a specific prediction about the perceptual effects of high contrast dazzle-type patterns: perception of an object's speed. This is a crucial determinant of capture success for predators that launch a ballistic strike at a moving prey or, in the military context, where a weapon is launched to intercept a moving target.

## Methods

Ethics statement: volunteers gave their informed written consent in accordance with the Declaration of Helsinki, and the experiments were approved by the Ethical Committee of the Department of Experimental Psychology, University of Bristol.

The experimental software was written in Matlab (The Mathworks Inc, Natick, MA), using the Psychophysics Toolbox extensions [19], [20] on a Macintosh G5 computer. All stimuli were viewed binocularly without a fixation point, 59 cm from the display, a linearised Trinitron Ultrascan P991 monitor (Sony, Japan) with a mean luminance of 47.7 cd/m^2^, a resolution of 1024×768 pixels and a refresh rate of 100 Hz).

**Figure 2:**
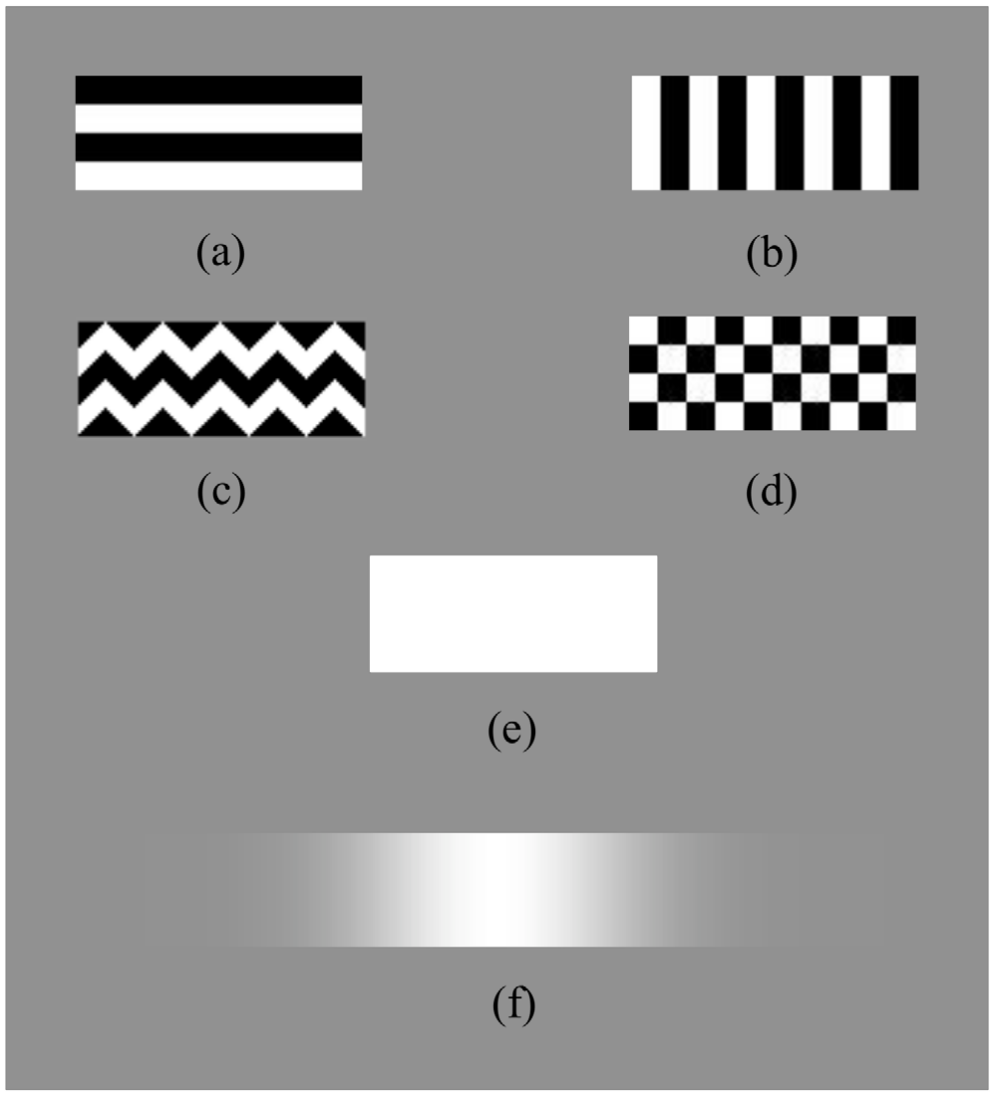
Stimuli. (a)–(e) standards, (f) comparison. (a) horizontal, (b) vertical, (c) zigzag, (d) check, (e) plain, (f) 1-D Gaussian. Stimuli (a)–(d) were displayed at two contrast levels (6.25% and 100%); stimulus (e) was displayed at maximum luminance (95.4 cd/m^2^); stimulus (f) ranged from mean luminance (47.7 m^2^) to maximum luminance (95.4 cd/m^2^).

On each trial, subjects were presented with a two temporal interval, binary choice task, and reported (via a keypad) which of the two stimuli moved more quickly. The speed of the standard stimulus was constant in a given block (either 3.33 or 20.0 deg/s), whilst that of the comparison stimulus was varied from trial to triaby the APE algorithm [21] in order to home in on the point of subjective equality. The comparison stimulus had a one-dimensional horizontal Gaussian luminance profile (fig.2f), which allowed for fine adjustment of its speed.

There were five standard stimuli (1.3×3.3 deg rectangles; fig.2), including a plain control stimulus (fig.2e), and only one stimulus type was displayed throughout any given block. All stimuli weredisplayed on a mean luminance background, and standard stimuli were displayed at either 6.25% or 100% contrast (blocked). All stimuli translated horizontally cross the screen for a randomly assigned duration (400–600 ms) in order to avoid distance travelled being used as a cue to stimulus speed. Two groups of 15 naïve subjects were tested at each of the contrast levels, with six subjects appearing in both groups.

## Results

**Figure 3:**
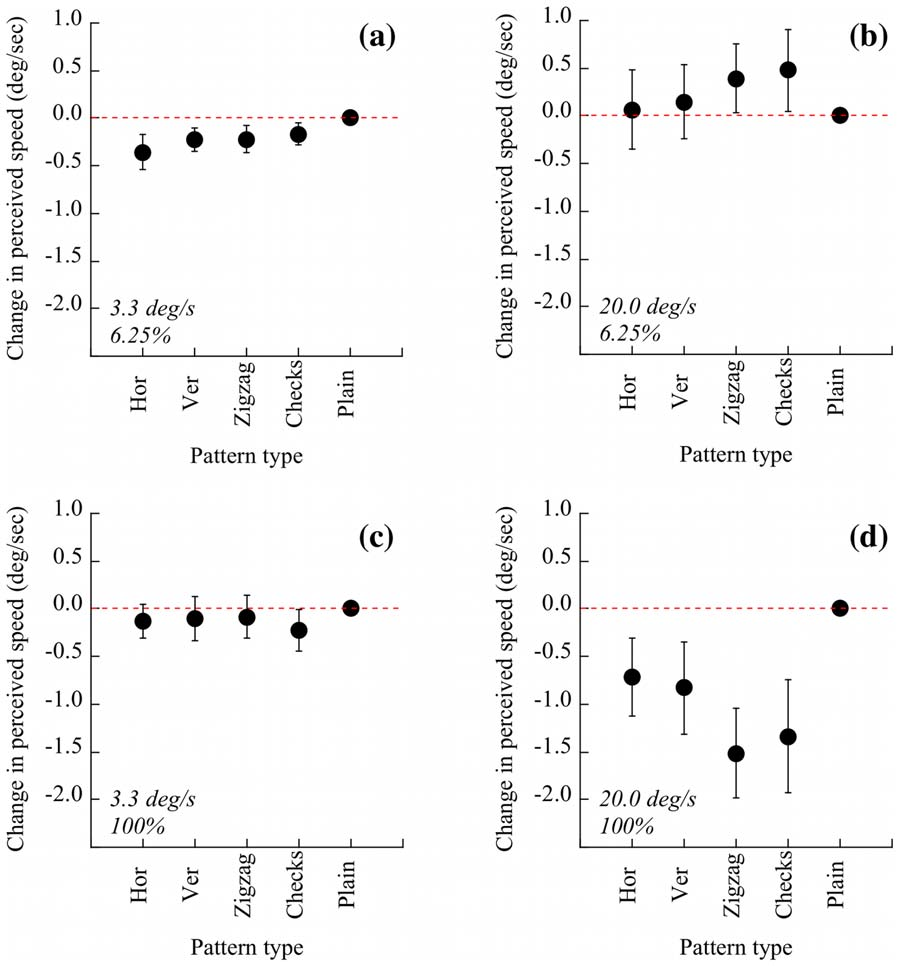
Data. The data are presented in terms of their deviation from the plain standard stimulus, which acts as a baseline measurement and is plotted as zero on the y-axis. (a) Low contrast, slow condition; (b) low contrast, fast condition; (c) high contrast, slow condition; (d) high contrast, fast condition. Error bars are ±1SEM.

Data are plotted as increments or decrements in perceived speed when compared with the plain pattern control stimulus (see fig.3). At low contrast and both speeds (figs.3a,b), there was no significant difference between perceived speed of any pattern when compared with the plain pattern. At high contrast and the slower speed (fig.3c), none of the textures differed from the plain pattern in perceived speed. But at the faster speed in the high contrast condition (fig.3d), there was a significant treatment effect with two textures perceived as moving slower than the plain pattern: zigzag (t_14_ = 3.23, p = 0.006) and check (t_14_ = 2.27, p = 0.04).

## Discussion

The experimental textures used were chosen to represent the typical range of components used in previously used dazzle camouflage: stripes, zigzags and checks. When moving quickly, two of the high contrast patterns tested caused a significant reduction in perceived speed of around 7%. These patterns - zigzags and checks - were two-dimensional, in contrast to the other, one-dimensional, patterns tested. Patterns which were less visible (low contrast) or slow moving had no effect on perceived speed; the former finding indicates that the effect is not simply due to texture *per se*, and implies that straightforward background-matching camouflage (which is generally lower contrast) would not produce a speed distortion: high contrast texture, as used for dazzle camouflage, is necessary.

The fast condition used (20 deg/s) translates into about 13 km/h (8 mph) at a distance of 10 m, and scales linearly upwards. So at the sort of ranges typical of naval warfare, we have no evidence that dazzle camouflage disguised the speed of ships in the two World Wars. Its efficacy in distorting other properties, such as size, shape, range and heading remains untested but, as outlined above, there are good reasons to suppose that these distortions occurred. A straightforward contrast effect on perceived speed [15] does not account for the data reported here: only two-dimensional patterns resulted in distorted speed perception, and only at high contrasts.

Thus dazzle patterns can distort perceived speed, if that speed is sufficiently high. As such, dazzle camouflage should be effective in situations where visual contact is still important: in nature and in low-tech battlefields. In the former case, dazzle may be one reason for high-contrast two-dimensional coloration (*e.g.* zebras). In the latter case, note that our experimental targets correspond approximately to a Land Rover at 70 m moving at 90 km/h. This is a typical distance between a rocket propelled grenade launcher and its target [22]. So if the target speed were sufficiently high, dazzle patterning should offer some protection from such devices. The effect size observed for check and zigzag patterns at this speed is an error of *c.*7% (fig.3d). An approximate calculation, based on the best available knowledge of the flight characteristics of a typical weapon, shows that the grenade takes around 0.5 s to reach a target at 70 m [22]; in 0.5 s a 90 kmh vehicle moves 12.5 m, and so a 7% error is about 90 cm. In other words, the missile would hit around 1 m behind where it was aimed, a difference which may be sufficient to prevent loss of life. Furthermore, the inherent variability of the effect with pattern, speed and contrast implies that using different patterns across vehicles will result in unpredictability: a good way to avoid easy compensation for the effect of the camouflage.
